# Associations of Genetic Variants Contributing to Gut Microbiota Composition in Immunoglobin A Nephropathy

**DOI:** 10.1128/mSystems.00819-20

**Published:** 2021-01-12

**Authors:** Jia-Wei He, Xu-Jie Zhou, Ya-Feng Li, Yan-Na Wang, Li-Jun Liu, Su-Fang Shi, Xiao-Hong Xin, Rong-Shan Li, Mario Falchi, Ji-Cheng Lv, Hong Zhang

**Affiliations:** a Renal Division, Peking University First Hospital, Beijing, People’s Republic of China; b Peking University Institute of Nephrology, Beijing, People’s Republic of China; c Key Laboratory of Renal Disease, Ministry of Health of China, Beijing, People’s Republic of China; d Key Laboratory of Chronic Kidney Disease Prevention and Treatment (Peking University), Ministry of Education, Beijing, People’s Republic of China; e Department of Nephrology, The Affiliated People’s Hospital of Shanxi Medical University, Taiyuan, Shanxi, People’s Republic of China; f Department of Twin Research and Genetic Epidemiology, King’s College London, London, United Kingdom; University of Hawaii at Manoa

**Keywords:** IgA nephropathy, genetics, microbiome quantitative trait loci, gut microbiota, *Dialister*

## Abstract

The gut microbiota has been implicated in immunoglobin A nephropathy (IgAN) because the intestinal immune response is assumed to be one of the disease triggers. Since the microbial composition is heritable, we hypothesize that genetic variants controlling gut microbiota composition may be associated with susceptibility to IgAN or clinical phenotypes. A total of 175 gut-microbiome-associated genetic variants were retrieved from the Genome-Wide Association Study (GWAS) Catalog. Genetic associations were examined in 1,511 patients with IgAN and 4,469 controls. Subphenotype associations and microbiome annotations were undertaken for a better understanding of how genes shaped phenotypes. Likely candidate microbes suggested in genetic associations were validated using 16S rRNA gene sequencing in two independent data sets with 119 patients with IgAN and 45 controls in total. Nine genetic variants were associated with susceptibility to IgAN. Risk genotypes of *LYZL1* were associated with higher serum levels of galactose-deficient IgA1 (Gd-IgA1). Other significant findings included the associations between the risk genotype of *SIPA1L3* and early age at onset, *PLTP* and worse kidney function, and AL365503.1 and more severe hematuria. Besides, risk genotypes of *LYZL1* and *SIPA1L3* were associated with decreased abundances of *Dialister* and *Bacilli*, respectively. Risk genotypes of *PLTP* and *AL365503.1* were associated with increased abundances of *Erysipelotrichaceae* and *Lachnobacterium*, respectively. 16S data validated a decreased tendency for *Dialister* and an increased tendency for *Erysipelotrichaceae* in IgAN. In this pilot study, our results provided preliminary evidence that the gut microbiota in IgAN was affected by host genetics and shed new light on candidate bacteria for future pathogenesis studies.

**IMPORTANCE** The gut microbiota and host genetics are implicated in the pathogenesis of IgAN. Recent studies have confirmed that microbial compositions are heritable (microbiome quantitative trait loci [QTL]). The relationship between host genetics and the microbiota and the role of the microbiota in IgAN are unclear. We retrieved the GWAS Catalog and associated microbiome QTL in IgAN, observing that nine genetic variants were associated with IgAN susceptibility and some clinical phenotypes. In a consistent way, the decreased abundance of *Dialister* was associated with higher serum levels of Gd-IgA1, and 16S rRNA gene analysis confirmed the decreased abundance of *Dialister* in IgAN. These data provided preliminary evidence that the gut microbiota in IgAN was affected by host genetics, which is a new strategy for future pathogenesis and intervention studies.

## Abstract (precis)

The gut microbiota and host genetics are implicated in the pathogenesis of IgAN. Recent studies have confirmed that microbial compositions are heritable (microbiome quantitative trait loci [QTL]).

## INTRODUCTION

Immunoglobulin A nephropathy (IgAN) is the most common form of primary glomerulonephritis, but its pathogenesis is not well understood. Current knowledge indicates that defects in IgA1 glycosylation that lead to the formation of immune complexes are at the center of its pathogenesis (1, 2). Genome-wide association studies (GWASs) in IgAN support that galactose-deficient IgA1 (Gd-IgA1) is heritable (3, 4), and they also suggest that some GWAS loci, such as *CARD9*, *TNFSF13*, and *PSMB8*, are shared among IgAN, inflammatory bowel disease (IBD), and bacterial infections (5). These findings highlight an important role for the intestinal immune response to mucosal pathogens in IgAN.

More recent studies directly checking the role of the gut microbiota indeed supported the role of the gut-kidney axis in IgAN, evidenced by studies in both human samples and mouse models (6–8). Functional studies suggested that bacterial infections, as well as some of their metabolites, could induce immune hyperresponsiveness, resulting in the overproduction of IgA and proinflammatory cytokines (9, 10). Moreover, the use of immunosuppressants targeting excessive mucosal immune responses and broad-spectrum antibiotics targeting gut microbes had shown effectiveness in treating IgAN (8, 11).

**FIG 1:**
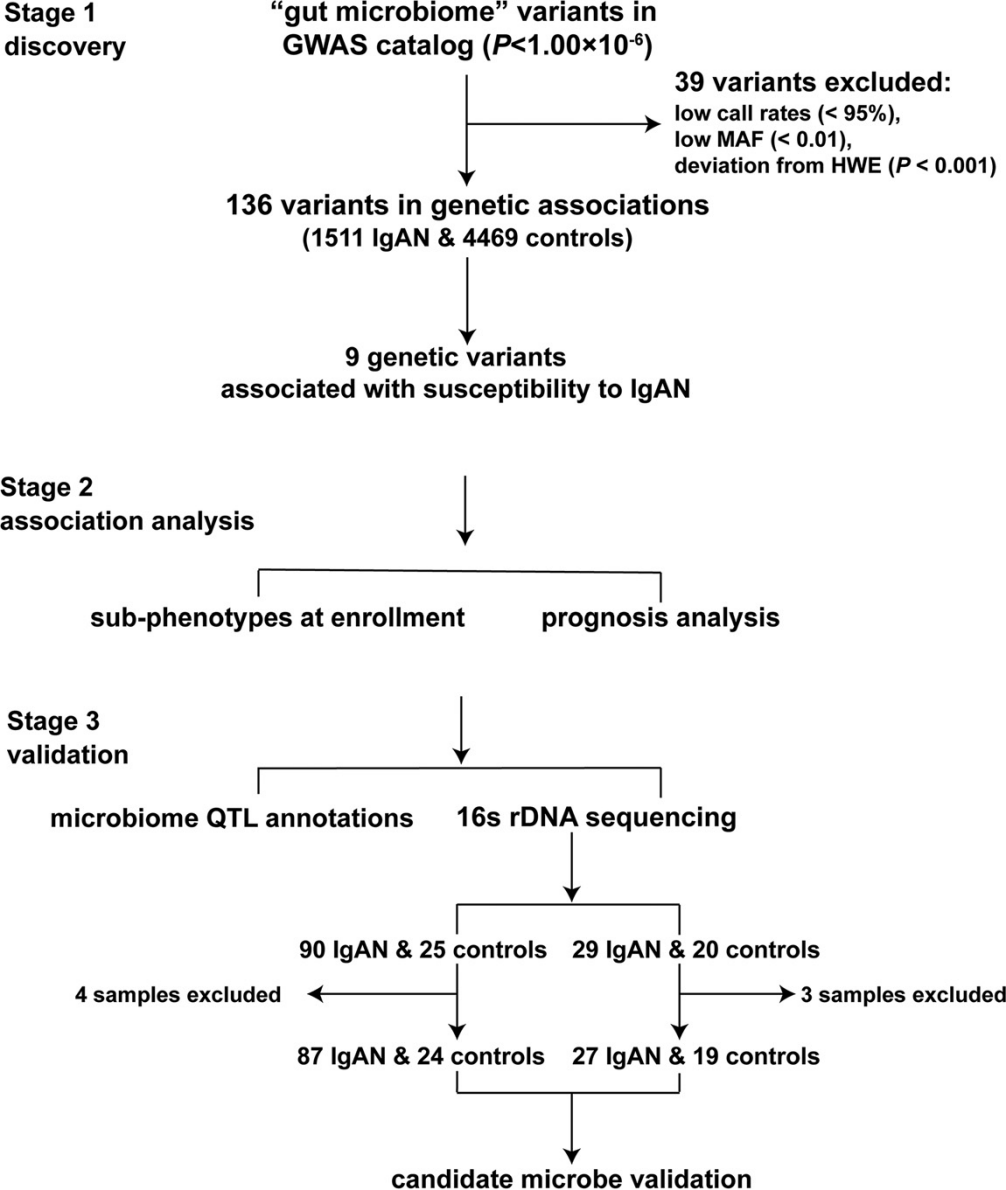
Flowchart of the current study. We use a three-step analysis strategy in this study. Abbreviations: HWE, Hardy-Weinberg equilibrium; MAF, minor allele frequency.

Similar to IBD, while it is known that both host genetics and the microbiome influence the development of the disease, how they precisely interact is less well understood (6, 12, 13). Recent studies have proven that the composition of the gut microbiota is heritable, and host-microbe interactions have a role in shaping the genetic architecture of IBD (14). Thus, in this pilot study, we aim to check the role of the microbiota in the etiology of IgAN in terms of host genetic susceptibility. The flowchart of the present study is shown in Fig. 1.

## RESULTS

### Genetic associations between genetic variants affecting the gut microbiome and susceptibility to IgAN.

As described in Materials and Methods, 136 genetic variants were left for genetic associations in this large cohort after quality control. Although we did not observe associations with genome-wide significance (5 × 10^−8^), nine variants were associated with IgAN with suggestive *P* values (Table 1). As two single nucleotide polymorphisms (SNPs) (rs1889714 and rs1248290) belonged to the same gene region and were in high linkage disequilibrium (*r*^2^ = 1.0), rs1889714, which had a lower *P* value, was selected for further analysis.

**TABLE 1:** The nine SNPs associated with IgAN with *P* values of <5 × 10^−2^^*a*^

CHR	SNP	Position (hg19)	Candidate gene(s)	Risk allele	RAF in cases (%)	RAF in controls (%)	*P* value	OR (95% CI)
6	rs3010562	167765251	*TTLL2*	T	52.85	49.49	1.39 × 10^−3^	1.14 (1.05, 1.24)
10	rs1889714	29388639	*LYZL1*, *LINC01517*	A	10.86	8.98	2.79 × 10^−3^	1.24 (1.08, 1.42)
20	rs6065904	44534651	*PLTP*	A	35.02	32.07	2.88 × 10^−3^	1.14 (1.05, 1.25)
6	rs9363741	68029041	AL365503.1, AL591004.1	G	17.21	15.01	4.05 × 10^−3^	1.18 (1.05, 1.31)
10	rs7083345	7031144	AL392086.3	C	86.54	84.38	5.19 × 10^−3^	1.19 (1.05, 1.34)
10	rs1248290	29386906	*LYZL1*, *LINC01517*	A	10.67	9.26	2.41 × 10^−2^	1.17 (1.02, 1.34)
18	rs11877825	10566404	*NAPG*, AP001099.1	G	79.43	77.49	2.86 × 10^−2^	1.12 (1.01, 1.24)
8	rs12541437	117918897	*RAD21-AS1*, *AARD*	T	40.04	37.86	3.55 × 10^−2^	1.10 (1.01, 1.19)
19	rs148330122	38520324	*SIPA1L3*	C	92.19	90.97	4.13 × 10^−2^	1.17 (1.01, 1.36)

a Abbreviations: CHR, chromosome; CI, confidence interval; OR, odds ratio; RAF, risk allele frequency.

### Genotype and subphenotype associations at enrollment.

**FIG 2:**
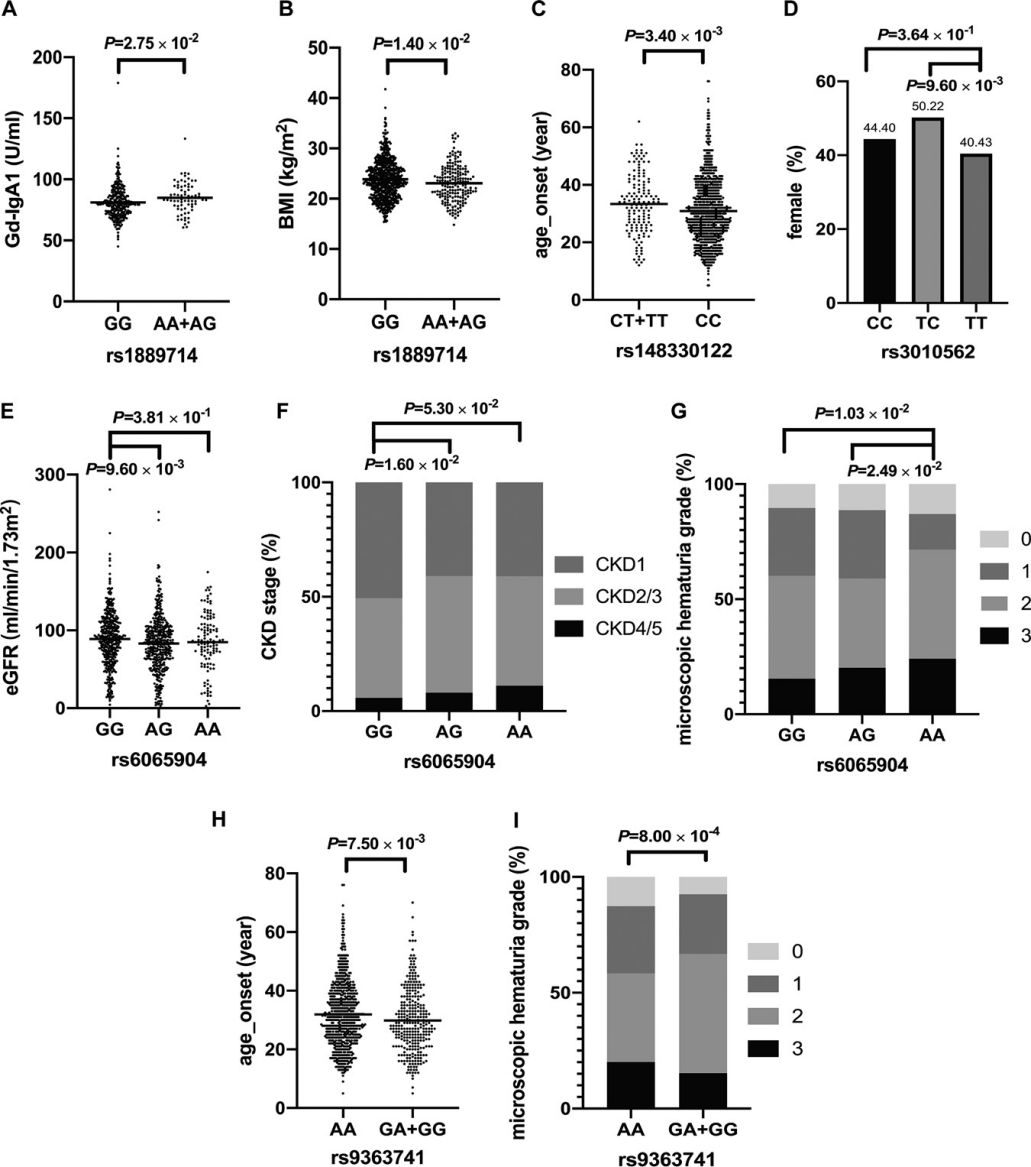
Associations between the genotypes and clinical subphenotypes of IgAN. (A) Patients with rs1889714-AA/AG genotypes (*n* = 75) had a higher serum level of Gd-IgA1 than did patients with the rs1889714-GG genotype (*n* = 294) (84.86 ± 12.27 U/ml in rs1889714-AA/AG versus 81.12 ± 13.26 U/ml in rs1889714-GG; the *P* value was determined by one-way ANOVA with Bartlett’s test for equal variances; the B test statistic is 0.407). (B) Patients with rs1889714-AA/AG genotypes (*n* = 191) had lower BMI than did patients with the rs1889714-GG genotype (*n* = 670) (23.08 ± 3.58 kg/m^2^ in rs1889714-AA/AG versus 23.85 ± 3.89 kg/m^2^ in rs1889714-GG; the *P* value was determined by one-way ANOVA with Bartlett’s test for equal variances; the B test statistic is 0.159). (C) Patients with the 148330122-CC genotype (*n* = 817) had lower age at onset than did patients with rs148330122-CT/TT genotypes (*n* = 144) (30.00 [interquartile range, 23.00, 38.00] years in rs148330122-CC versus 33.50 [26.00, 41.00] years in rs148330122-CT/TT; the *P* value was determined by a two-tailed Wilcoxon rank sum test). (D) The proportions of female patients with the rs3010562-TT genotype (*n* = 282), the rs3010562-TC genotype (*n* = 454), and the rs3010562-CC (*n* = 232) genotype were 40.43%, 50.22%, and 44.40%, respectively (the *P* value was determined by Pearson’s chi-squared test). (E) The eGFR was lower in patients with the rs6065904-AG genotype (*n* = 425) than in patients with the rs6065904-GG genotype (*n* = 417) (83.99 [62.95, 102.91] ml/min/1.73 m^2^ versus 90.36 [67.10, 111.20] ml/min/1.73 m^2^; the *P* value was determined by a two-tailed Wilcoxon rank sum test). (F) Patients with the rs6065904-AG genotype (*n* = 425) had worse kidney function than did patients with the rs6065904-GG genotype (*n* = 417) (CKD1, 2/3, and 4/5, 40.94%, 51.06%, and 8.00%, respectively, in the rs6065904-AG genotype versus 50.60%, 43.65%, and 5.76%, respectively, in rs6065904-GG; the *P* value was determined by Pearson’s chi-squared test). (G) Patients with the rs6065904-AA genotype (*n* = 116) had worse microscopic hematuria than did patients with rs6065904-AG (*n* = 425) and patients with the rs6065904-GG genotype (*n* = 415) (the *P* value was determined by Pearson’s chi-squared test). (H) Patients with rs9363741-GG/GA genotypes (*n* = 300) had a younger age at onset than did patients with the rs9363741-AA genotype (*n* = 669) (28.25 [22.00, 36.75] years in rs9363741-GG/GA versus 31.00 [24.00, 39.00] years in rs9363741-AA; the *P* value was determined by a two-tailed Wilcoxon rank sum test). (I) Patients with the rs9363741-GG/GA genotypes (*n* = 295) had worse microscopic hematuria than did patients with the rs9363741-AA genotype (*n* = 666) (MH grades 0, 1, 2, and 3, 7.46%, 25.76%, 51.53%, and 15.25%, respectively, in rs9363741-GG/GA versus 12.61%, 28.98%, 38.29%, and 20.12%, respectively, in rs9363741-AA; the *P* value was determined by Pearson’s chi-squared test).

Next, we analyzed the associations between the risk variants and clinical subphenotypes of IgAN (Fig. 2; see also Table S1 in the supplemental material) under additive models unless genotypic counts were <10. The risk genotypes of *LYZL1* rs1889714-AA/AG were associated with higher serum levels of Gd-IgA1 and lower body mass index (BMI) (Fig. 2A and B). The risk genotype of *SIPA1L3* rs148330122-CC was associated with a younger age at onset (Fig. 2C). The risk genotype of *TTLL2* rs3010562-TT was associated with male predominance (Fig. 2D).

10.1128/mSystems.00819-20.1 TABLE S1 Detailed genotype and subphenotype associations among the eight genetic variants and clinical subphenotypes of IgAN. Download Table S1, XLSX file, 0.02 MB. Copyright © 2021 He et al. 2021 He et al. This content is distributed under the terms of the Creative Commons Attribution 4.0 International license.

Risk genotypes of *PLTP* rs6065904-AA/AG were associated with worse kidney function and more severe microscopic hematuria (MH) (Fig. 2E to G). The risk genotypes of *AL365503.1* rs9363741-GG/GA were associated with a younger age at onset and more severe hematuria (Fig. 2H and I). Of note, the associated clinical subphenotypes, such as age, sex, and BMI, had already been suggested to be strongly associated with the composition of the gut microbiota (15, 16).

### Prognosis associations.

**FIG 3:**
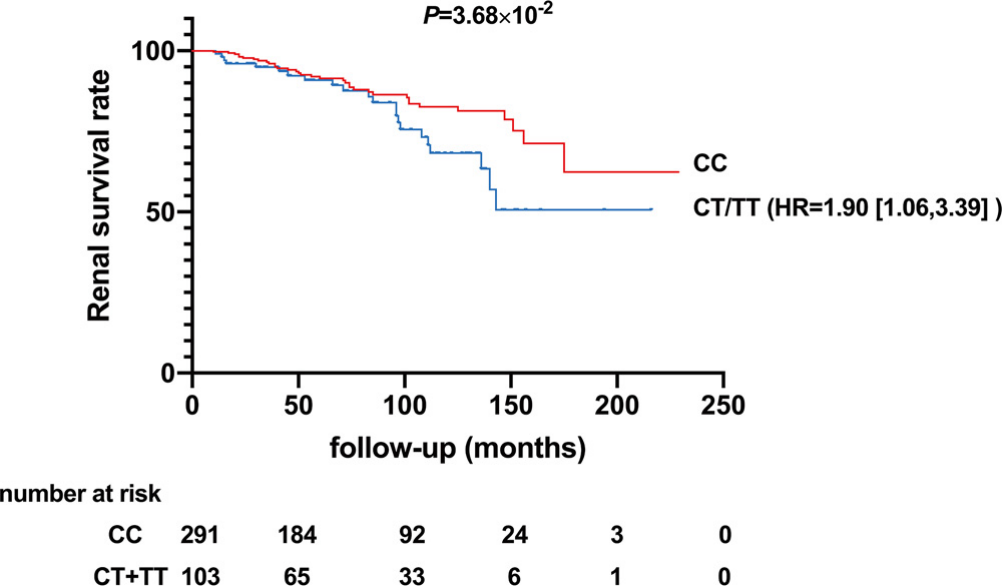
Cumulative renal survival rate in 409 patients with IgAN with regular follow-up information. Kaplan-Meier analysis showed that the cumulative renal survival rate was significantly lower in patients with rs7083345-CT/TT genotypes (the *P* value was determined by a log rank test). HR, hazard ratio.

In the current cohort, 409 patients with IgAN were regularly monitored. The median duration of follow-up was 6.5 years. We observed a significant association between genotypes of *AL392086.3* and the risk of end-stage renal disease (ESRD), which occurred in 22 out of 103 patients (21.36%) with rs7083345-CT/TT genotypes and 35 out of 291 patients (12.03%) with the CC genotype. Kaplan-Meier analysis showed that the cumulative renal survival rate was significantly lower in patients with rs7083345-CT/TT genotypes (Fig. 3).

Consistent with previous reports (17, 18), the univariate analysis indicated that renal function, Gd-IgA1, and proteinuria were the prognostic factors in IgAN. Moreover, the risk genotype of rs7083345 was observed to be an additional prognostic factor. In the multivariate Cox regression analysis, rs7083345-CT/TT genotypes, lower estimated glomerular filtration rate (eGFR), and higher time-averaged proteinuria were independently associated with poorer renal prognosis (Table 2).

**TABLE 2:** Univariate and multivariate analyses of risk factors associated with ESRD in patients with IgAN^*a*^

Factor	Univariate analysis		Multivariate analysis	
	HR (95% CI)	*P* value	HR (95% CI)	*P* value
rs7083345-TT/CT	**1.75 (1.03, 2.99)**	**3.90 × 10^−2^**	**1.90 (1.06, 3.39)**	**3.00 × 10^−2^**
Age (yrs)	1.01 (0.98, 1.03)	5.85 × 10^−1^	0.99 (0.96, 1.03)	7.03 × 10^−1^
Sex (female)	0.67 (0.40, 1.12)	1.26 × 10^−1^	1.03 (0.57, 1.86)	9.10 × 10^−1^
Serum creatinine (μmol/liter)	**1.02 (1.01, 1.02)**	**<1.00 × 10^−3^**		
eGFR (ml/min/1.73 m^2^)	**0.97 (0.96, 0.98)**	**<1.00 × 10^−3^**	**0.96 (0.95, 0.97)**	**<1.00 × 10^−3^**
Gd-IgA1 (U/ml)	**1.02 (1.00, 1.03)**	**4.90 × 10^−2^**	1.01 (0.99, 1.03)	2.33 × 10^−1^
Serum IgA (g/liter)	0.93 (0.75, 1.15)	4.99 × 10^−1^		
Time-averaged proteinuria (g/g)	**2.03 (1.67, 2.48)**	**<1.00 × 10^−3^**	**2.52 (1.89, 3.35)**	**<1.00 × 10^−3^**
Urine protein excretion (g/24 h)	**1.20 (1.09, 1.31)**	**<1.00 × 10^−3^**		
Serum C3 (g/liter)	0.43 (0.11, 1.70)	2.29 × 10^−1^		
CKD stage				
2/3	**2.95 (1.51, 5.76)**	**2.00 × 10^−3^**		
4/5	**24.58 (9.77, 61.80)**	**<1.00 × 10^−3^**		

a *P* values which are <0.05 are highlighted by boldface type. HR, hazard ratio.

### Microbiome QTL annotations.

By data mining of the previous results on microbiome quantitative trait loci (QTL) (Table 3), it was shown that the risk genotypes of rs1889714 (*LYZL1*) in IgAN were negatively correlated with the abundance of *Dialister*, which has been reported to be significantly decreased in patients with IgA vasculitis or Crohn’s disease (19, 20). The risk genotypes of rs148330122 (*SIPA1L3*) and rs7083345 (*AL392086.3*) in IgAN were reported to be negatively correlated with the abundance of *Bacilli*, and the risk genotype of rs3010562 (*TTLL2*) in IgAN was reported to be negatively associated with the abundance of *Anaerofilum.* These microbiota members were suggested to be beneficial in maintaining intestinal homeostasis (21, 22).

**TABLE 3:** Microbiome QTL annotations

Genetic variant/risk allele in present study	Genetic variant/risk allele in GWAS Catalog	Variant annotation	Associated microbiome^*a*^	Beta	*P* value	GWAS Catalog accession no.
rs1889714-A	rs1889714-A	Intergenic variant	*s_Dialister_invisus*	0.41-U decrease	6.00 × 10^−9^	GCST003855
rs1248290-A	rs1248290-A	Intergenic variant	*g_Dialister*	0.40-U decrease	1.00 × 10^−8^	GCST003855
rs148330122-C	rs148330122-C	Intron variant	*c_Bacilli*	0.48-U decrease	1.00 × 10^−9^	GCST003875
rs7083345-C	rs7083345-T	Intron variant	*c_Bacilli*	0.25-U increase	3.00 × 10^−10^	GCST003875
rs3010562-T	rs3010562-G	Intron variant	*g_Anaerofilum*	0.67-U increase	3.00 × 10^−7^	GCST003222
rs6065904-A	rs6065904-A	Intron variant	*g_Erysipelotrichaceae*	0.19-U increase	4.00 × 10^−6^	GCST003854
rs11877825-G	rs11877825-G	Regulatory region variant	*f_Erysipelotrichaceae*	0.27-U decrease	3.00 × 10^−11^	GCST003875
rs9363741-G	rs9363741-G	Intergenic variant	*g_Lachnobacterium*	0.86-U increase	5.00 × 10^−7^	GCST003222
rs12541437-T	rs12541437-T	Intergenic variant	*g_Lachnobacterium*	0.60-U increase	2.00 × 10^−6^	GCST003223

a Abbreviations: *c_*, class; *f_*, family; *g_*, genus; *s_*, species.

On the other hand, the risk genotype of rs6065904 (*PLTP*) in IgAN was positively associated with the abundance of *Erysipelotrichaceae*, and risk genotypes of rs9363741 (*AL365503.1*) and rs12541437 (*RAD21-AS1*) in IgAN were reported to be positively associated with the abundance of *Lachnobacterium* (14). Both *Erysipelotrichaceae* and *Lachnobacterium* were suggested to be detrimental to gut disease (23, 24).

### Gut microbiota in patients with IgAN.

We included two independent data sets to check the diversity and structure of the microbial community using the 16S rRNA technique. After quality control by removing the abnormal samples due to low connectivity (<2.5), we had 87 cases with IgAN (age, 38.78 ± 11.45 years; 47.13% female; BMI, 23.87 ± 3.37 kg/m^2^) and 24 matched healthy controls (age, 35.79 ± 8.19 years; 50.00% female; BMI, 22.64 ± 2.81 kg/m^2^) in the first data set. In the second data set, we had 27 cases with IgAN (age, 44.68 ± 12.65 years; 20% female) and 19 healthy controls (age, 31.32 ± 10.59 years; 57.89% female) in 16S rRNA analysis without selection.

**FIG 4:**
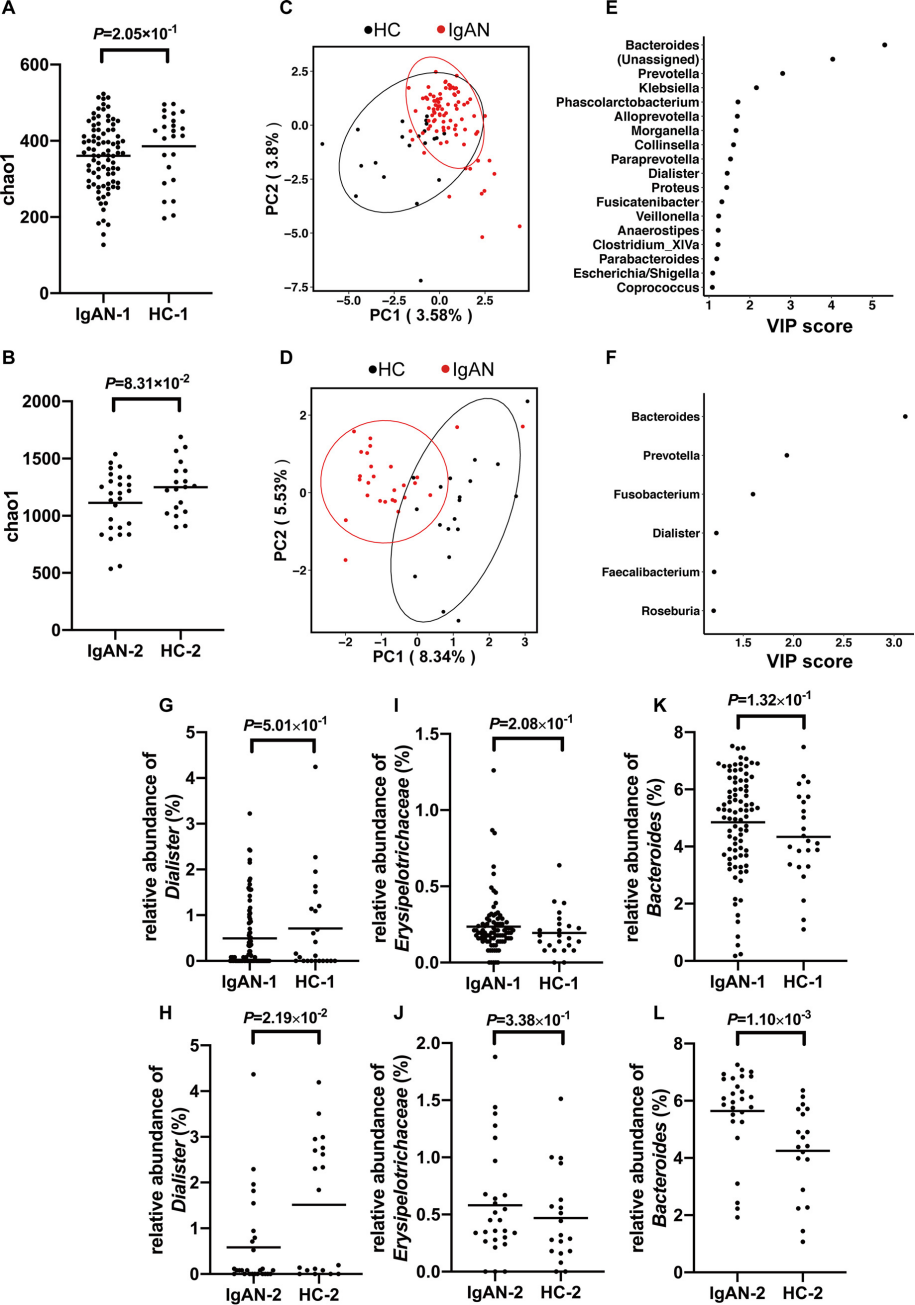
16S rRNA gene analysis in two independent IgAN data sets. (A and B) There was no significant difference in alpha diversity (Chao1) between cases with IgAN and controls in two independent data sets (the *P* value was determined by a two-tailed Wilcoxon rank sum test). (C and D) PLS-DA score plot of species abundance in samples from individuals with IgAN and healthy controls (HC). It showed a distinct clustering pattern between groups (permutational multivariate analysis of variance with the Bray-Curtis distance metric was used to assess the significance of differences between the two groups [*P = *4.5 × 10^−2^] for data set 1 [C] and for data set 2 [D]). (E and F) VIP scores of PLS-DA. A taxon with a VIP score of ≥1 was considered necessary for the group’s discrimination. The VIP scores of the candidate genus *Dialister* were 1.45 and 1.23, and the VIP scores of the genus *Bacteroides* were 5.30 and 3.11, for data set 1 (E) and data set 2 (F), respectively. (G and H) The relative abundance of the genus *Dialister* tended to decrease in cases compared to healthy controls. (I and J) The relative abundance of the family *Erysipelotrichaceae* tended to be enriched in patients with IgAN. (K and L) The relative abundance of the genus *Bacteroides* tended to increase in patients with IgAN. For panels G to L, all the data are expressed as the square root of the relative abundance of the specific bacterium. *P* values were determined by a two-tailed Wilcoxon rank sum test. PC, principal component.

The alpha diversity of bacterial communities was evaluated according to the Chao1 diversity index (Fig. 4A and B). Generally, there was no statistical significance between patients with IgAN and controls in terms of alpha diversity. Partial least-squares discriminant analysis (PLS-DA), a supervised learning method, showed a distinct clustering pattern between samples from cases with IgAN and healthy controls in both data sets (Fig. 4C and D). For both data sets, the top one microbe, which contributed most significantly to differentiate cases from controls, was the same. The variable importance in projection (VIP) scores for *Bacteroides* were 5.30 and 3.11, respectively (Fig. 4E and F).

In confirmation of clues suggested by the genetic associations, *Dialister* was also observed to play a role in group separation (VIP = 1.45 and 1.23, respectively). To check the difference at the genus level using Wilcoxon rank sum tests, as shown in Fig. 4G and H, the relative abundance of *Dialister* showed a decreased tendency in IgAN patients, and the relative abundance of *Erysipelotrichaceae* showed an increased tendency (Fig. 4I and J). For the microbe with the top VIP score, *Bacteroides* tended to increase in both data sets (Fig. 4K and L). For *Bacilli*, *Anaerofilum*, and *Lachnobacterium*, which were associated with the former genetic associations, no output data were available due to the limited richness of 16S rRNA gene sequencing. The complete results about the relative abundances at the genus level in two independent data sets are shown in Tables S2 and S3 in the supplemental material.

10.1128/mSystems.00819-20.2 TABLE S2 16S rRNA gene data on bacterial taxonomy and relative abundance in the first data set. Download Table S2, XLSX file, 0.1 MB. Copyright © 2021 He et al. 2021 He et al. This content is distributed under the terms of the Creative Commons Attribution 4.0 International license.

10.1128/mSystems.00819-20.3 TABLE S3 16S rRNA gene data on bacterial taxonomy and relative abundance in the second data set. Download Table S3, XLSX file, 0.02 MB. Copyright © 2021 He et al. 2021 He et al. This content is distributed under the terms of the Creative Commons Attribution 4.0 International license.

**FIG 5:**
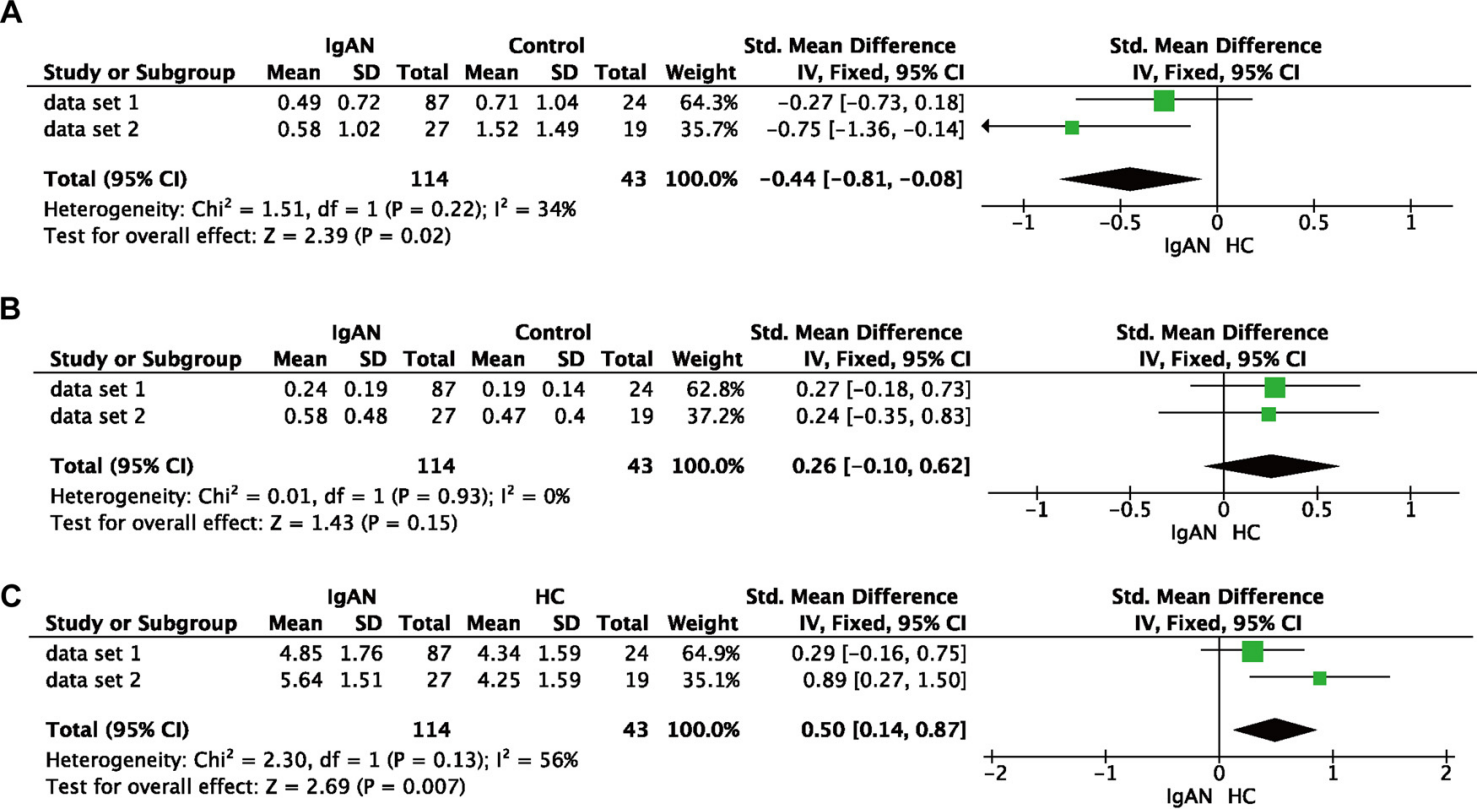
Meta-analysis of the relative abundances of *Dialister* (A), *Erysipelotrichaceae* (B), and *Bacteroides* (C) in two data sets. The relative abundance of *Dialister* was reduced in patients with IgAN (*P = *2.00 × 10^−2^), while the relative abundance of *Bacteroides* was increased in patients with IgAN (*P = *7.00 × 10^−3^). However, the relative abundance of *Erysipelotrichaceae* showed no significant difference (*P = *1.50 × 10^−1^). Abbreviations: CI, confidence interval; IV, inverse variance.

To further confirm the differential abundance, we conducted a meta-analysis for the two data sets (Fig. 5). There was moderate statistical heterogeneity (30% to 60%), but the forest plots showed consistent associations, with a decreased tendency for *Dialister* and an increased tendency for *Bacteroides* in IgAN.

## DISCUSSION

The microbial composition was associated with multiple traits (e.g., age, sex, and BMI) and diseases (e.g., cardiovascular disease and IBD) (15, 25, 26). Meanwhile, microbial compositions could be affected by multiple factors such as the environment and host genetics. Genetic variants associated with the microbiome are defined as microbiome QTL. Recent studies have identified microbiome QTL in human diseases, including IBD, cancer, heart disease, and meningitis (27, 28). These microbiome QTL can be regulated by genes involved in microbiome-related pathways, including the immune system, food metabolism, and drug-related systems.

In the current study, we systemically checked the microbiome QTL in reported GWASs. We further investigated associations between reported microbiome QTL and the susceptibility to and severity of IgAN. In a large cohort with 1,511 patients with IgAN and 4,469 controls, we observed that 9 SNPs were associated with susceptibility to IgAN. Genotype-phenotype associations between risk alleles and disease subtypes may provide insight into disease etiology and mechanisms. Intriguingly, these risk variants were observed to be associated with subphenotypes of IgAN, i.e., early age at onset, elevated Gd-IgA1 levels, severe hematuria, and advanced chronic kidney disease (CKD) stage., and in a concordant way, specific risk genotypes in IgAN were associated with decreased abundances of potentially beneficial microbes and increased abundances of potentially harmful microbes, as reported for IBD. Furthermore, one microbiome-associated SNP was also independently associated with renal outcome in IgAN. Along with a gene-centered study, we further checked the validity of the candidate microbiota members by 16S rRNA gene sequencing in two independent data sets to increase statistical power. G*Power 3.1.9.6 software was used to calculate the posterior effect size index using the asymptotic relative efficiency (ARE) method (29). The effect sizes were between 0.25 and 0.30. To estimate the power of the Wilcoxon test for a given *F* value with the ARE method, we scaled the sample size with the corresponding ARE value. The calculated powers in cohort 1 and cohort 2 were 0.22 and 0.14, respectively. After meta-analysis, the power was 0.34 for the combined samples. According to the results from the two data sets, we confirmed that both microbiota richness/evenness and the abundance of *Dialister* were decreased in IgAN. However, this candidate microbe was not the most significant in IgAN. In addition, according to the VIP score, we observed that *Bacteroides* contributed the most in differentiating IgAN from controls. It has been proven that *Bacteroides* can impact fecal IgA levels (30). However, as *Bacteroides* was not included in our candidate microbiome QTL, we cannot rule out the role of host genetic impacts. Therefore, by a multistage association strategy in this pilot study, we tentatively confirmed that host genetics had impacts on the gut microbiota, which played a role in both disease susceptibility and severity.

For the function and pathogenesis of the microbiota in IgAN, recent studies suggested that changes of the gut microbiota could alter IgA-mediated immunity, and differences in IgA binding to bacteria have been linked to IBD (31), which shares some similarities in etiology and is also a common comorbidity with IgAN. It is widely recognized that IgA plays a pivotal and special role in IgAN, and our data supported the hypothesis that individuals with IgAN have an altered gut microbiota, which was somewhat genetically determined. Interestingly, the *LYZL1* risk genotypes, which were *Dialister* related, were associated with an elevated level of Gd-IgA1. The relative abundance of the bacterial genus *Dialister* was reported to be significantly decreased in children with IgA vasculitis (19), in which an increased level of Gd-IgA1 was a common pathogenic mechanism, as in IgAN. Besides, in mouse studies, the c-type lysozyme gene *Lyzl1* exhibited antibacterial activity (32), and mouse models targeting *LYZL1* and *Dialister* will be of interest. Studies have highlighted the role of the gut microbiome in IgA or Gd-IgA1 immunity. Specific microbes or metabolites are known to be involved in the class switch of B cells and the production of IgA (7, 30). This study may have significance in future translational medicine. First, we observed that host genetics might affect gut microbiota composition. This genetic information might contribute to risk stratification. Since genetics, instead of diet, were unchanged, we might check whether the tested people were genetically susceptible to abnormal bacterial composition, IgA immunity, and IgAN, especially in risk populations or risk families. Second, a precise link between genetics and the gut microbiota would shed new light on disease pathogenesis. It was speculated that both the mucosal immune response and the gut microbiome participated in the pathogenesis of IgAN. But the precise role of the gut microbiota in mediating the gut-kidney dialog in IgAN has not been clarified. Last but not least, targeting specific bacteria or certain metabolites would have therapeutic significance in IgAN. Thus, future studies integrating host genetics and microbiota may shed some light on precision medicine, possibly by targeting IgA immunity and infections.

Despite a large-scale genetic study on microbiome QTL in IgAN, we should note some limitations. First, there were still no widespread microbiota-related studies in IgAN, not to mention an IgAN disease-specific microbiome QTL study. To guarantee ≥80% power to detect a 1.2-fold-increased risk, we may need at least 2,366 cases and 7,000 controls (see Table S4 in the supplemental material), and we cannot rule out the possibility that other genetic variants had a higher magnitude of risk effects in IgAN, i.e., *Bacteroides*-associated variants in IgAN. Second, fecal 16S rRNA gene sequencing was limited in precision in microbiota differentiation. Meanwhile, our study did not differentiate between progressor and nonprogressor IgAN patients, whose gut microbiome profiles were different (6). However, consistent with our results, their study also observed decreased alpha diversity and key microbe changes (Bacteroides coprocola, Bacteroides faecis, and *Dialister* sp.) in IgAN. Thus, a prospective study using metagenomic sequencing to check the relationship between the gut microbiota and disease progression is warranted. Third, we tried our best to minimize the impact of confounding factors, including body weight, BMI, gender, alcohol intake, tobacco use, and diet. Those who were vegetarians or had dietary bias were excluded before enrollment. However, we had to acknowledge that the proportions of some constituents of the diet, such as salt or fatty acids, were not controlled in our cohorts (33–35). Fourth, all the associations may not bear multiple corrections. With 8 independent variants and 13 clinical variables, a conservative Bonferroni threshold to declare a significant association would be 4.81 × 10^−4^ (0.05/8/13), whereas it may indicate that the functional impact of the gut microbiota was not very high since the occurrence and progression of IgAN were the products of the complex interactions between genetics and the environment. Fifth, both genetic association analysis and 16S rRNA sequencing were conducted in participants with the same genetic background from the same living district (Han Chinese ethnicity). Replication from different ethnicities was the gold standard for evaluating the size and reliability of a genetic finding. As a first attempt to explore potential associations with any available trait, the current data were tempting, but more widespread replications focusing on these eight SNPs would be needed in the future.

10.1128/mSystems.00819-20.4 TABLE S4 Power calculation to estimate the number of patients required with ≥80% power. Download Table S4, XLSX file, 0.01 MB. Copyright © 2021 He et al. 2021 He et al. This content is distributed under the terms of the Creative Commons Attribution 4.0 International license.

In conclusion, the first pilot microbiome QTL genetic study in IgAN showed that eight independent genetic variants were associated with both the susceptibility to and severity of IgAN. Along with feces microbe confirmation, our data indicated the decreased abundance of potentially beneficial microbes in IgAN, which might shed some light on future interventions.

## MATERIALS AND METHODS

### SNP selection.

We selected the potentially gut-microbiome-associated genetic variants identified by GWASs by searching the NHGRI GWAS Catalog database as of 5 March 2019 using the keyword “gut microbiome” (36). Finally, we selected 175 genetic variants associated with the gut microbiome (*P* < 1.00 × 10^−6^). These genetic variants were associated with alpha/beta diversity, gut microbiota taxa and their relative abundance (37–39). Here, we provide all the related information about these genetic variants in Table S5 in the supplemental material.

10.1128/mSystems.00819-20.5 TABLE S5 Retrieved information for the 136 genetic variants investigated in the current study. Download Table S5, XLSX file, 0.03 MB. Copyright © 2021 He et al. 2021 He et al. This content is distributed under the terms of the Creative Commons Attribution 4.0 International license.

### Genotyping and study populations.

For genetic associations, a total of 5,980 participants, comprising 1,511 cases with IgAN and 4,469 healthy controls of Chinese ancestry, were recruited from Peking University First Hospital. The diagnosis of IgAN was confirmed by kidney biopsy with immunofluorescence studies for IgA deposits. Cases with secondary IgAN, such as systematic lupus erythematosus, rheumatic disease, or IgA vasculitis, were excluded. Ethnically and geographically matched healthy controls were voluntarily recruited.

Peripheral blood samples were collected from participants using the anticoagulant EDTA. We obtained the genomic DNA from peripheral blood leukocytes using the salting-out method. Genotyping was performed by using the MassArray system from Sequenom (Compass Biotech, Beijing, China). SNPs with low call rates (<95%), low minor allele frequencies (MAFs) (<0.01), or deviation from Hardy-Weinberg equilibrium (*P < *0.001) were excluded.

### Clinical variables.

Demographics and clinical data at the time of renal biopsy check included age, gender, BMI, blood pressure, urinary sediment microscopy, 24-h urine protein excretion, serum IgA, complement component 3, and serum creatinine. Microscopic hematuria was defined as ≥3 red blood cells (RBCs) per high-power microscopic field (HPF) in three consecutive urinalyses (40) and was graded as 0 (0 to 3 RBCs/HPF), 1 (3 to 15 RBCs/HPF), 2 (15 to 100 RBCs/HPF), or 3 (>100 RBCs/HPF). Time-averaged proteinuria was defined as the mean of every 6 months of proteinuria measurements. Kidney disease severity was classified into five stages according to the eGFR based on KDIGO guidelines (41) and was grouped as mild (chronic kidney disease stage 1 [CKD1]), moderate (CKD2 and 3), and severe (CKD4 and 5). Gd-IgA1 was detected in 388 cases by a lectin enzyme-linked immunosorbent assay (ELISA) as previously described (42).

Among the cases enrolled, 409 patients were regularly monitored. Clinical outcome of ESRD was defined as an eGFR of <15 ml/min/1.73 m^2^ or the application of renal replacement therapies, including hemodialysis, peritoneal dialysis, or renal transplantation.

### 16S rRNA gene sequencing.

16S rRNA gene sequencing was conducted to confirm the likely involved microbes suggested by the genetic associations. In total, we recruited 119 cases with IgAN and 45 healthy controls. Patients with ESRD, secondary IgAN, IBD, and type 2 diabetes mellitus were excluded. For both patients and healthy controls, those who reported the use of antibiotics, microbial agents, or immunosuppressants within 8 weeks before entry were excluded. Besides, those who were vegetarians or had dietary bias were also excluded. Healthy controls had no gastrointestinal diseases.

The propensity score method was used to adjust for known confounding factors, including age, sex, and BMI. Thus, the enrolled participants were divided into two groups. The first data set consisted of 90 cases with IgAN and 25 age/sex/BMI-matched healthy controls, and the second data set consisted of the rest of the 29 cases with IgAN and 20 healthy controls without selection. Fecal samples were deemed biologically representative specimens because of their noninvasion and convenience. The fecal samples from each participant were collected in the hospital and immediately stored at −80°C until 16S rRNA gene sequencing.

Fecal genomic DNA was extracted using the QIAamp Fast DNA stool minikit (catalog number 51604; Qiagen). The targeted V3-V4 hypervariable region of the bacterial 16S rRNA genes was amplified by PCR using the primers 341F (5′-CCTACGGGRSGCAGCAG-3′) and 806R (5′-GGACTACVVGGGTATCTAATC-3′). Amplicons were extracted from 2% agarose gels and purified using the AxyPrep DNA gel extraction kit (catalog number AP-GX-50; Axygen Biosciences) and quantified using Qubit2.0 (Invitrogen, MA, USA). After the preparation of the library, sequencing was performed on a HiSeq platform to generate paired-end reads of 250 bp (Illumina, CA, USA) for the first data set. The same protocol as the one described above was adopted for the second data set, and the tags were sequenced on the MiSeqDx platform (Illumina, CA, USA).

### Data preprocessing.

Consistent methods were used to process all qualified data from the two data sets. The output reads were processed using USEARCH v.10 and in-house scripts (43, 44). The quality of the paired-end Illumina reads was checked by FastQC v.0.11.5 (45) and processed in the following steps by VSEARCH: joining of paired-end reads and relabeling of sequencing names, removal of barcodes and primers and filtering of low-quality reads (*Q* < 20), and finding of nonredundant reads. Unique reads were denoised into amplicon sequence variants (ASVs)/operational taxonomic units (OTUs). The representative sequences were picked by UPARSEH (46). The OTU table was generated by USEARCH. The taxonomy of the representative sequences was classified with the RDP classifier (47). Analysis of the differential OTU abundance and taxa was performed using the edgeR v3.26.8 package in R v.3.6.1. We subsample (“rarefy”) an OTU table to a fixed number of reads per sample using random subsampling without replacement. Besides, the abnormal observations were filtered if the connectivity was <2.5, using the Weighted Correlation Network Analysis (WGCNA) package (48).

Alpha diversity was evaluated by the Chao1 diversity index. PLS-DA was used to reveal taxonomic changes in different groups, and VIP scores were used to rank the abilities of different taxa to discriminate groups (49). Wilcoxon rank sum tests (two tailed) were conducted to detect differences in relative abundances between the two groups.

### Ethics statement.

The study complied with the Declaration of Helsinki and was approved by the Ethics Committee of Peking University First Hospital (Institutional Review Board [IRB] approval numbers 2013[548] and 2019[76]). Written informed consent was obtained from all the patients involved in both genotyping and 16S rRNA gene sequencing.

### Statistical analysis.

A two-tailed *P* value of <0.05 was considered statistically significant. Genetic association analysis was performed using PLINK-1.9 (50). Subphenotype associations were taken under the additive models at the genotypic level. To ensure the stability of the analysis, only if the sample count for the risk homozygous genotype was <10, a dominant model was considered.

Continuous variables in this study were compared using an unpaired *t* test or analysis of variance (ANOVA) between groups if the variables were normally distributed; otherwise, a Mann-Whitney U test or a Kruskal-Wallis test was performed. Categorical variables were compared using the chi-square test or Fisher’s exact test. Cumulative renal survival rates were calculated according to the Kaplan-Meier method. Univariate and multivariate Cox regression analyses were used to evaluate the risk of ESRD. The statistical analysis was performed with SPSS 26.0 software (SPSS Inc., USA).

As two different sequencing platforms were adopted in this study, we conducted a meta-analysis based on group differences rather than directly combining demultiplexed sequences. Raw data were processed into a normal distribution by calculating the square root of the relative abundance of the specific microbe. Also, further measures of the group differences were processed by Review Manager (version 5.4; Cochrane Library) under the fixed-effect model, and the degree of statistical heterogeneity was quantified by an *I*^2^ test.
